# Characterization and parameters for the effectiveness of a UV‐C device for infection control in dental clinics

**DOI:** 10.1111/php.70065

**Published:** 2026-01-18

**Authors:** Kátia Cristiane Hall, Everton Granemann Souza, Chiara das Dores do Nascimento, Marcel Luiz Basso, Mário Lúcio Moreira, Carla Lucía David Pena, Wellington Luiz Oliveira da Rosa, Rafael Guerra Lund, Evandro Piva

**Affiliations:** ^1^ Graduate Program in Dentistry (PPGO) School of Dentistry Federal University of Pelotas (UFPel) Pelotas RS Brazil; ^2^ Graduate Program in Electronics and Computer Engineering (MEEC) Catholic University of Pelotas (UCPel) Pelotas RS Brazil; ^3^ Graduate Program in Materials Science and Engineering (PPGCEM) Federal University of Pelotas (UFPel) Pelotas RS Brazil; ^4^ Institute of Physics and Mathematics (IFM), Graduate Program in Physics (PPGFis) Federal University of Pelotas (UFPel) Capão do Leão RS Brazil

**Keywords:** antimicrobial resistance, colorimetric dosimetry, dental clinic, infection control, UV‐C disinfection, UVGI

## Abstract

Healthcare‐associated infections (HAIs) remain a significant concern in dental settings, especially in light of increasing antimicrobial resistance. This study aimed to characterize and evaluate the efficacy of an ultraviolet C (UV‐C) disinfection device specifically developed as an additional resource for dental clinics and healthcare settings. Colorimetric dosimeters were strategically distributed at different points and vertical levels to monitor UV‐C radiation distribution in the environment. The emission spectrum and radiation intensity were measured using spectral measurement techniques, while microbiological analyses were conducted on seven commonly encountered surfaces in dental offices using mannitol salt agar selective for *Staphylococcus aureus* and *Staphylococcus epidermidis*. The results revealed a significant reduction in colony‐forming units (CFUs), exceeding 90% at most sampling points (*p* < 0.05). An exponential falloff of irradiance with distance from the UV‐C source was observed, highlighting the importance of proper positioning of the equipment. Notably, the UV‐C device proved effective even on surfaces with higher microbial loads, such as the armrest of the dental chair. The findings demonstrate that both time and distance significantly affect disinfection efficacy, and that purpose‐built UV‐C devices are viable as complementary tools to conventional chemical cleaning and disinfection protocols.

## Graphical abstract

Purpose‐built UV‐C device achieves over 90% disinfection of high‐touch dental clinic surfaces.

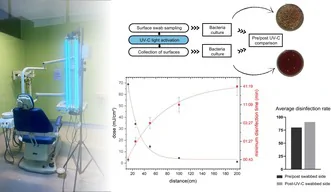

**Abbreviations**
CDC: Centers for Disease Control and Prevention
CFU: colony‐forming unit(s)
DNA: deoxyribonucleic acid
E_90_
: irradiance at 90% of the steady‐state value
EN: European standard (European Norm)
E_ss_
: steady‐state irradiance
HAI(s): healthcare‐associated infection(s)
ISO: International Organization for Standardization
MSA: mannitol salt agar
RNA: ribonucleic acid
*S. aureus*
: *Staphylococcus aureus*
*S. epidermidis*
: *Staphylococcus epidermidis*
UV/Vis: ultraviolet/visible
UVA: ultraviolet A
UVB: ultraviolet B
UV‐C (UVC): ultraviolet C
WHO: World Health Organization

## INTRODUCTION

The prevalence of healthcare‐associated infections (HAIs), particularly in hospitals and dental settings, remains a significant global concern, further exacerbated by the rise of antimicrobial resistance.^1^ In addition, HAIs impose a substantial financial burden on healthcare systems, resulting in prolonged hospitalizations, the need for additional treatments, and an increase in antibiotic use. It is estimated that these infections may cost billions of dollars annually to both public and private healthcare systems, representing a significant economic challenge.

Within this broader context, dental clinics represent a critical environment for infection control. Recent evidence indicates that, even after routine cleaning and disinfection, dental clinic surfaces may remain contaminated with opportunistic and potentially multidrug‐resistant bacteria, such as *Staphylococcus aureus*, which are frequently implicated in HAIs.^2^ These residual contaminants highlight the limitations of manual protocols in achieving uniform decontamination and underscore the need for adjunctive disinfection strategies capable of reaching areas that are difficult to clean manually and reducing the likelihood of cross‐contamination in dental environments.^3^

Sterilization and chemical disinfection are well‐established methods in the control of these infections, playing a fundamental role in biosafety protocols.^4^ Autoclaving remains the gold standard for sterilizing heat‐resistant instruments, effectively eliminating all microbial forms, including bacterial spores.^5^ Chemical disinfectants, such as alcohol, hydrogen peroxide, and quaternary ammonium compounds, are widely used for surface decontamination in hospitals and dental clinics.^6^ While effective, these agents have limitations related to application time, material compatibility, and potential toxicity when used for prolonged periods, as well as environmental impacts due to the disposal of chemical waste.^4^

Frequently touched surfaces and equipment in these environments act as reservoirs for multidrug‐resistant microorganisms, facilitating pathogen transmission. Therefore, enhancing disinfection methods or implementing additional protocols such as ultraviolet C (UV‐C) irradiation is crucial to reducing infection risks, requiring the integration of complementary technologies to support traditional cleaning practices.^3^

Considering these challenges, UV‐C radiation has gained attention as an overlay method since it is a complementary alternative for disinfecting surfaces and medical equipment, enhancing traditional cleaning and sterilization methods without replacing them.^7^ UV‐C irradiation is recommended by international health organizations, such as the Centers for Disease Control and Prevention (CDC) and the World Health Organization (WHO), as an additional infection control measure in healthcare settings.^8^ With wavelengths between 200 and 280 nm, UV‐C light inactivates viruses and bacteria by damaging their DNA and RNA, making it highly effective even against multidrug‐resistant pathogens.^9^

Besides reaching surfaces and equipment that are difficult to disinfect manually, UV‐C offers a chemical‐free method that does not leave residual disinfectants, making it also an environmentally sustainable alternative.^9^, ^10^ However, its disinfection performance is strongly influenced by surface geometry, material properties, shadowing and the distance between the lamp and the target area.^11^, ^12^, ^13^, ^14^ Nonuniform irradiation and shielded regions are recurrently reported in hospital and dental settings and can markedly reduce germicidal effectiveness, emphasizing the need for site‐specific dosimetry and positioning strategies.

In this context, the main contribution of the present study is an integrated methodological framework for evaluating UV‐C systems in complex clinical geometries. A key strength of the approach is the experimental design that combines point‐by‐point colorimetric dosimetry with microbiological sampling at the same locations, enabling a direct correlation between delivered UV‐C dose and CFU reduction.

The surface subdivision strategy (halving each object, using triplicate subareas, and pairing “swabbed” vs. “not swabbed before UV‐C” regions) provides, to our knowledge, a novel and quantitative way to assess any mechanical effect of swabbing on the observed disinfection efficacy. Combined with full spectral characterization of the lamps, detailed dose–distance measurements, and spatially resolved dosimetry throughout the operatory (walls, ceiling, and clinically relevant surfaces), this framework allows us to link how much UV‐C is actually delivered across the room to how much microbial inactivation is achieved on each sampled surface when the device is used as a complementary antimicrobial protocol in a dental operatory.

## MATERIALS AND METHODS

### UV‐C disinfection system and spectral characterization

**FIGURE 1:**
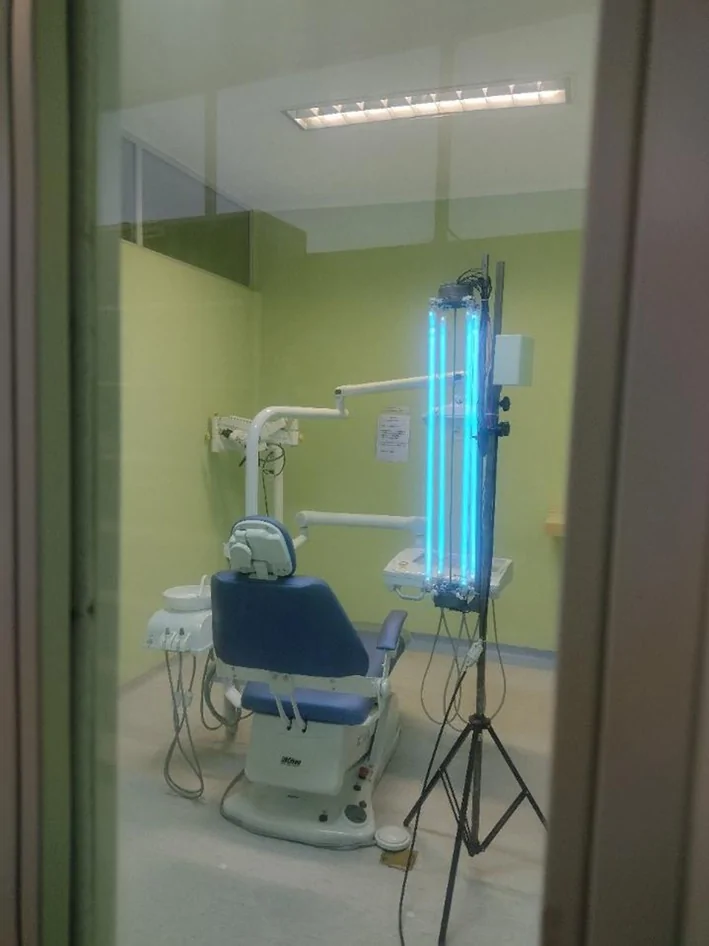
UV‐C disinfection tower designed for dental clinic environments. The system comprises four germicidal low‐pressure mercury UV‐C lamps arranged vertically and symmetrically around a central support structure to promote uniform irradiance in the surrounding area. Electrical connections and ballast components are located at the rear of the system.

The disinfection equipment used in the work is a noncommercial prototype designed and assembled by our research group. It consisted of four low‐pressure UV‐C germicidal lamps (model T8 30GER, SCT Comercial Elétrica, Brazil) with a nominal power of 30 W per lamp and a length of 90 cm (total electrical power 120 W) vertically mounted on a 1.80 m tall mobile metallic tower (Figure 1).

The tower was mounted on locking casters so that it could be positioned beside the dental chair and adjusted to vary the distance between the radiation source and the target surfaces. The lamps were driven by electronic ballasts and controlled by a programmable timer switch (Sonoff, Itead, China), which allowed preset exposure times and automatic shut‐off; in this study, a 5‐min exposure was used for all irradiation cycles. All disinfection procedures were performed in an unoccupied room with the door closed and a warning sign indicating UV‐C in operation. The operator positioned the tower and performed microbiological sampling only while the lamps were off, wearing a lab coat, disposable gloves, face mask, and protective eyewear, in accordance with the institutional biosafety and UV‐safety guidelines. This configuration enabled reproducible control of exposure time and lamp‐surface distance, which was essential to assess UV‐C disinfection performance and user safety under different materials and spatial arrangements in the dental office.

The emission spectrum of the UV‐C lamps was characterized using a UV/Vis Flex+ spectrometer (Sarspec, Portugal) to analyze the wavelength range, with particular emphasis on the 200–280 nm band, which corresponds to the germicidal region.^15^, ^16^ The measurements were taken at a distance of 25 cm using an optical fiber with a core diameter of 200 μm. The emission spectrum was obtained using Light Scan software (version 1.1.17), which enabled the identification of the predominant germicidal wavelengths. The relationship between the UV‐C radiation dose, distance, and exposure time, obtained in Section “Spectral Characterization and Dose–Distance Behavior of the UV‐C Disinfection System,” was determined using an LMS‐ 6000UV (Lisun, China) spectroradiometer. Measurements were conducted at five fixed distances: 25, 50, 75, 100, and 150 cm from UV‐C lamps with a fixed exposure time of 1 min. The radiation dose (mJ/cm^2^) was calculated based on the readings obtained, enabling the construction of an exponential decay curve of light intensity as a function of distance.

### Test surfaces and environmental conditions

To assess the effectiveness of UV‐C radiation on different surfaces, seven materials commonly found in dental offices were selected, including both porous and nonporous surfaces. The analyzed materials included stainless steel tables and shelves, laminated wood surfaces, and vinyl coatings of the dental chair. The selection of these materials allowed for the investigation of UV‐C radiation interaction with substrates exhibiting different reflection and absorption indices. The environmental conditions of the dental operatory were monitored during the experiments. The average air temperature was approximately 24°C, and the relative humidity was around 70%. During all UV‐C exposure cycles, the ventilation and air‐conditioning systems of the dental operatory remained turned off to prevent external interference in the experiment.

All procedures were carried out in a fully operational dental operatory, where the sampling points were surfaces routinely exposed to bioaerosols generated during clinical procedures such as ultrasonic scaling and high‐speed handpiece use. Before each experimental session, the clinic's standard preshift hygiene protocol was followed: all countertops, the dental chair, and the dental unit were disinfected with 70% ethanol, and PVC barriers were applied to high‐touch areas. No patients were treated during the experimental period.

UV‐C irradiation was performed immediately after this routine cleaning and before the start of any clinical activity, so the bioaerosols and settled microorganisms on the sampled surfaces represent the residual contamination that normally persists under routine practice, while ensuring equivalent and standardized baseline conditions across all surfaces.

### Microbiological sampling and UV‐C dose mapping with colorimetric films

Microbiological assays were performed in a standard dental clinic room (4.10 × 2.75 m). Surface samples were collected from seven predefined sampling points (P1–P7), corresponding to a laminated wood corner table (P1), the upper stainless‐steel shelf of the instrument table (P2), the lower stainless‐steel shelf (P3), a stainless‐steel auxiliary table (P4), the vinyl‐covered dental chair seat (P5), the vinyl‐covered dental chair armrest (P6), and a laminated wood back table (P7).

**FIGURE 2:**
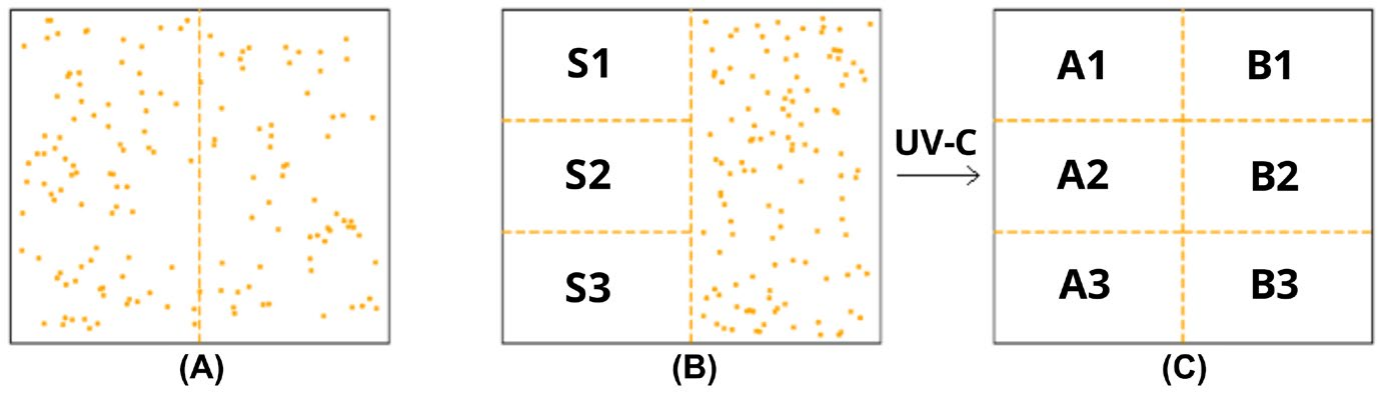
Schematic representation of the surface subdivision strategy used for microbiological sampling. (A) Initial situation: the tabletop is considered to have an approximately uniform distribution of microorganisms and is conceptually divided into two halves. (B) Before UV‐C irradiation, only the left half of the surface is sampled. This half is subdivided into three horizontal subareas (S1, S2, S3), each collected with an independent swab, while the right half remains untouched and represents an adjacent control area. (C) After a single 5‐min UV‐C exposure, both halves are subdivided into three horizontal subareas (A1–A3 on the left and B1–B3 on the right), to enable paired comparison, in triplicates, between regions that were previously swabbed and regions that had not been swabbed before irradiation.

To provide an internal control for the mechanical effect of swabbing, we developed a new sampling strategy based on surface subdivision. First, each sampling point was divided into two symmetrical halves, assuming an approximately homogeneous distribution of microorganisms over the surface, as illustrated in Figure 2A for a single sampling point.

At each point, only one half was swabbed before UV‐C exposure (Figure 2B). Microorganisms were carefully collected with a sterile swab and immediately streaked onto sterile Petri dishes containing mannitol salt agar (MSA; Kasvi, Ref. K25‐1062, Brazil), a selective medium for *S. aureus* and *Staphylococcus epidermidis*, pathogens frequently found in clinical environments. After the UV‐C irradiation cycle (Figure 2C), both halves of each sampling point were swabbed and inoculated onto fresh MSA plates. This ensures robust statistics, since all samples were collected in triplicate, as indicated in Figure 2B,C.

Subsequently, all plates were incubated at 37°C for 48 h, and the number of colony‐forming units (CFU) was quantified. The total interval encompassing microorganism collection before irradiation, the UV‐C exposure, and microorganism collection after irradiation at all points remained well below 10 min. This design allowed a paired comparison between areas that had been swabbed before irradiation and adjacent areas that had not, thereby assessing whether the swabbing procedure itself influenced the apparent disinfection efficiency. In the Results section, these conditions are reported as averaged values under the labels: “before irradiation (control),” “after irradiation (previously collected),” and “after irradiation (not collected).”

In parallel with microbiological sampling, colorimetric dosimeters (UVSCALE, Fujifilm, Tokyo, Japan) were used to map the spatial distribution of UV‐C dose. These films provide a time‐integrated dose readout rather than instantaneous irradiance, integrating all incident UV‐C fluence over the exposure period. All films were prepared according to the same procedure and had identical exposed areas of 1 × 1 cm, ensuring a constant dosimetric area at all monitored locations. An initial exploratory mapping was performed by distributing dosimeters over the room envelope (12 units on the walls and 15 on the ceiling), with the UV‐C tower operating for 5 min near the dental chair (excluding warm‐up time). The coloration of each film was converted into dose (mJ/cm^2^) by comparison with a laboratory‐fabricated calibration template previously validated against spectroradiometric measurements.^17^, ^18^ The corresponding results are presented in Section “In situ dosimetric assessment on floor and ceiling surfaces”.

For the main experiment, dosimeters were positioned exactly at the seven microbiological sampling points (P1–P7), enabling direct correlation between local UV‐C dose and CFU reduction, while an additional set of wall and ceiling films was retained to monitor dose propagation throughout the operatory.

### Statistical analysis

Microbiological data (CFU counts) were summarized as mean ± standard deviation for each sampling point. Because CFU distributions were highly skewed and the sample size was small (*n* = seven points), paired comparisons were performed using the Wilcoxon signed‐rank test rather than parametric tests. For each surface, the point‐wise mean CFU before UV‐C exposure was paired with the corresponding point‐wise mean CFU measured on the previously swabbed half (“clean side”) and on the nonswabbed half (“unclean side”) after the 5‐min irradiation cycle. In all cases, the null hypothesis was that there was no systematic change in CFU counts (i.e., a median difference of zero between the “before” and “after” measurements).

Reduction rates were computed for each point as, *R* = (Before irradiation − After irradiation)/Before irradiation, and are reported as mean ± standard error across the seven sampling points. The standard error of *R* at each point was estimated by propagation of uncertainty from the within‐point variability of the “before” and “after” measurements. All tests were two‐sided, and a significance level of α = 0.05 was adopted to determine statistical significance.

## RESULTS AND DISCUSSION

### Spectral characterization and dose–distance behavior of the UV‐C disinfection system

**FIGURE 3:**
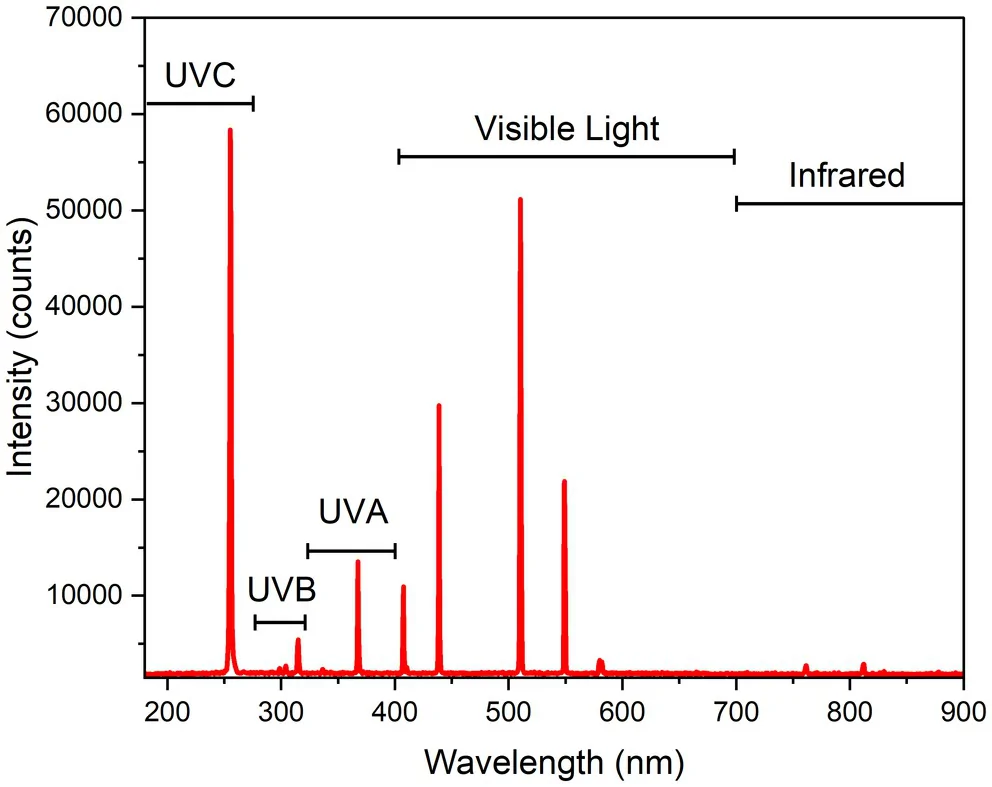
Peak of the predominant wavelengths emitted by the UV‐C lamps of the studied disinfection tower.

UV‐Vis optical spectroscopy was employed to characterize the ultraviolet light emitted by the disinfection tower, allowing for the determination of intensity across the entire UV‐C wavelength range. As shown in Figure 3, the lamps exhibited a prominent emission peak at 254 nm, a wavelength widely recognized for its germicidal efficacy due to its ability to damage microbial nucleic acids. No emission was detected in the 185 nm region (not shown), which is the wavelength primarily responsible for ozone generation from molecular oxygen. This confirms that the UV‐C lamps operate as an ozone‐free system, consistent with low‐pressure mercury lamps whose quartz envelope is doped to block ozone‐producing wavelengths.

In addition to the main UV‐C peak, secondary emissions were also detected in the UV‐B and UV‐A regions. However, as reported in earlier studies^19^, ^20^ radiation in these bands contributes negligibly to disinfection: UVA and visible light are essentially nongermicidal, and although UVB can exhibit some germicidal activity, its intensity in our system was too low to be relevant.

**FIGURE 4:**
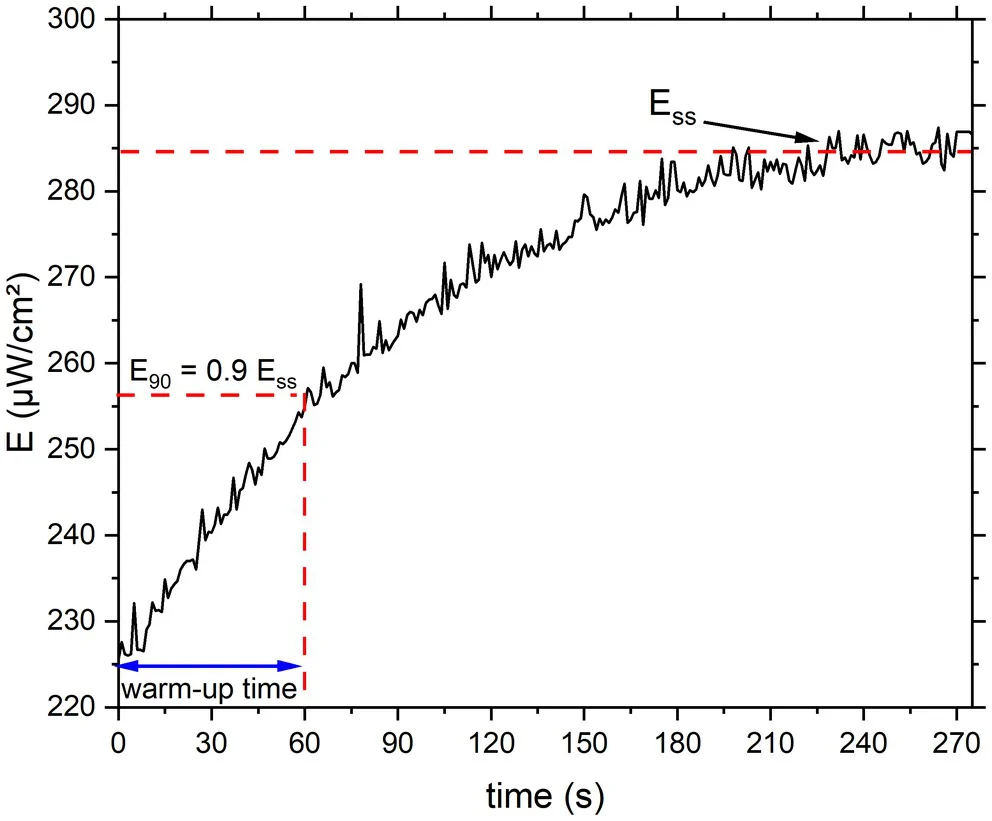
Time‐resolved irradiance of the low‐pressure UV‐C disinfection tower at 25 cm from a cold start. The solid black line shows the irradiance *E*(*t*) as a function of time, from switch‐on to the steady‐state regime. The upper red dashed line indicates the steady‐state irradiance *E*_ss_, while the lower red dashed line marks *E*_90_ = 0.9 *E*_ss_. The vertical red dashed line and the blue horizontal arrow highlight the warm‐up time, that is, the time required for *E*(*t*) to reach 90% of *E*_ss_ (approximately 60 s).

Figure 4 shows the time‐resolved irradiance measurements used to evaluate the temporal stability of the UV‐C output. The irradiance was recorded at a fixed distance of 25 cm from the source. Immediately after switch‐on, it was approximately 225 μW/cm^2^ and gradually increased to a steady‐state level (*E*_ss_) of about 280 μW/cm^2^ over the 270 s monitoring period.

Following a commonly used criterion,^21^ the warm‐up time was defined as the time required for the irradiance to reach 90% of *E*_ss_ (E_90_ = 0.9 *E*_ss_). In our system, this threshold was reached after roughly 60 s, at which point the irradiance was already close to 257 μW/cm^2^ (approximately 92% of *E*_ss_). The subsequent rise in output represented only a minor fraction of the final value; when integrated over the 5 min exposure used in the in situ experiments, the difference between the actual warm‐up curve and an ideal, instantly stabilized lamp corresponds to a reduction of only about 2% in the delivered dose.

**FIGURE 5:**
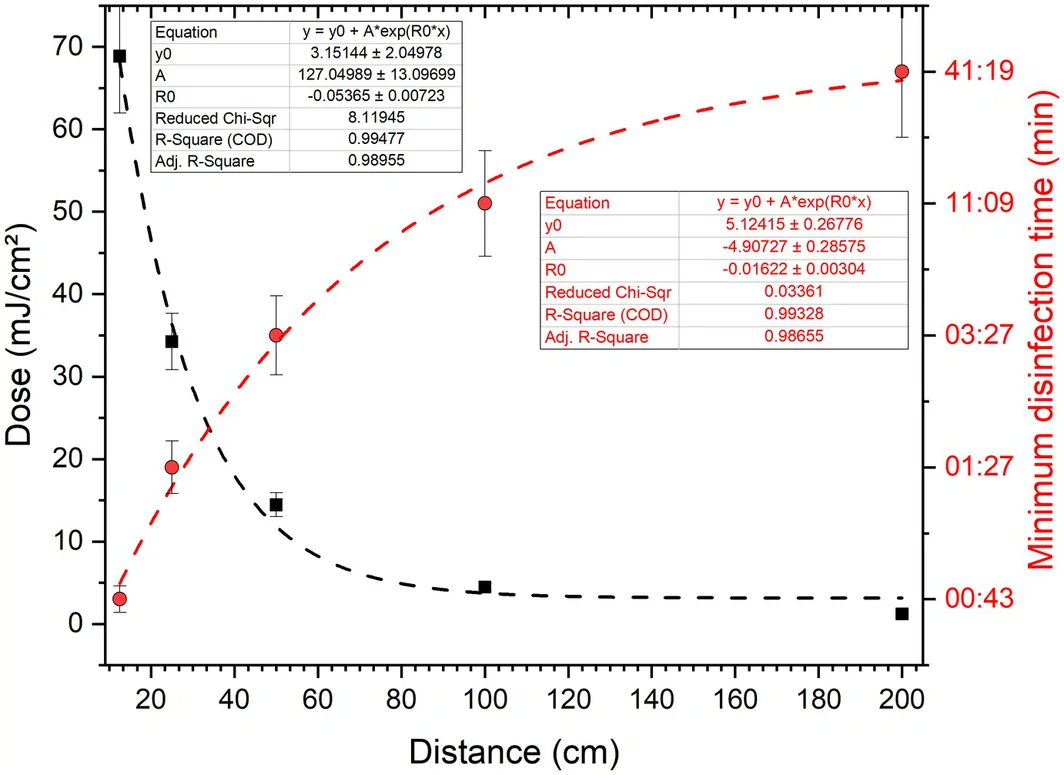
Dose versus distance and time to reach a 50 mJ/cm^2^ dose (target as lethal for multidrug‐resistant pathogenic bacteria).

Figure 5 presents the UV‐C dose versus distance and the exposure time required to reach 50 mJ/cm^2^, a benchmark often sufficient to inactivate multidrug‐resistant bacteria. This target is commonly used in efficacy/safety assessments of UV‐C devices (ISO 15858:2016; EN 17272:2020)^19^, ^20^ and aligns with fluence concepts discussed by Bolton et al.^21^

Measured after a 1 min warm‐up of the disinfection tower, the dose is ∼70 mJ/cm^2^ at 20 cm and falls to ∼2 mJ/cm^2^ at 200 cm. The corresponding times to reach 50 mJ/cm^2^ are plotted in red.

Although the inverse square law theoretically governs irradiance decay from a point source in free space,^20^ our experimental data showed a better fit to an exponential decay model. This deviation is attributed to real‐world factors such as lamp geometry, air absorption of UV‐C photons,^17^ and nonideal beam collimation. Similar behavior has been described by Kowalski and Mazur,^15^ supporting the use of empirical exponential models for greater accuracy in indoor dosimetry.

The second curve (red dots), which follows an increasing exponential trend, represents the exposure time required to reach the 50 mJ/cm^2^ threshold. At 20 cm from the UV‐C source, this dose was reached in approximately 43 s. At 200 cm, however, the required exposure time exceeded 41 min. This reinforces the critical importance of proximity for effective disinfection. The exponential relationship between distance and disinfection time confirms that UV‐C efficacy is strongly dependent on the proximity of the light source to the target surface.^18^ This finding is particularly relevant for clinical applications in dentistry, where complex geometries and multiple surfaces require precise planning of UV‐C placement and exposure time.^22^

### In situ dosimetric assessment on floor and ceiling surfaces

In addition, the spatial distribution of the dose was assessed using both colorimetric films and bacterial cultures placed at the same locations, which increased the accuracy of the results, unlike most studies in the literature that rely solely on biological markers based on bacteria.^21^

**FIGURE 6:**
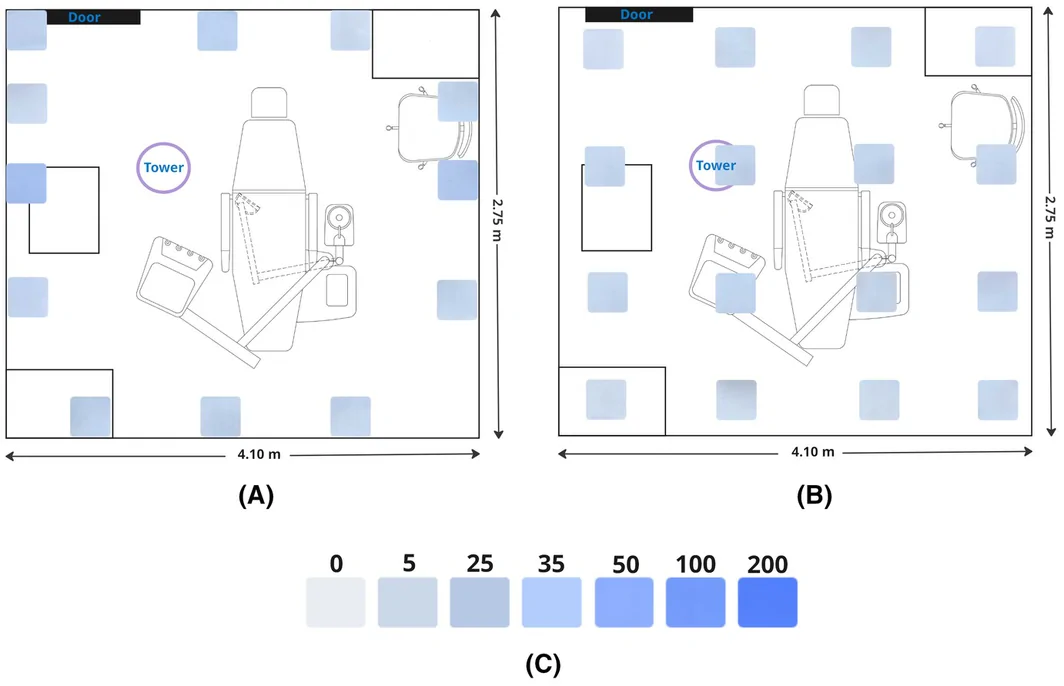
(A) Distribution of dosimeters mounted on the ceiling to assess vertical reach and uniformity of the radiation field. (B) Spatial distribution of colorimetric dosimeters placed on the side walls to evaluate lateral UV‐C propagation. (C) Calibration template used for UV‐C dose quantification. The figure displays colorimetric dosimeters with varying color intensities corresponding to different radiation doses, enabling visual interpretation of UV‐C exposure levels. The numbers above each sample are expressed in mJ/cm^2^ and represent the absorbed dose measured by the dosimeters.

To assess dose propagation and determine the optimal positioning of the UV‐C tower, colorimetric dosimeters were initially placed on the walls and ceiling of the dental clinic prior to defining the measurement points, as detailed in section “Microbiological Sampling and UV‐C Dose Mapping with Colorimetric Films.” For this test, the tower was positioned near the dental chair and operated for 5 min, as shown in Figure 6A,B. The dose estimation was performed by comparing the colorimetric response to a laboratory‐fabricated calibration template, whose design was validated using spectroradiometric analysis, as shown in Figure 6C.^18^, ^23^

By observing the dosimeters colors on the ceiling, Figure 5B, variations in UV‐C dose distribution were observed. In central regions directly exposed to the light source, the delivered dose is between 25 and 30 mJ/cm^2^, indicating adequate and consistent irradiation. These areas, situated near the vertical axis of the tower, received direct exposure, which accounts for the higher radiation values recorded.

In contrast, peripheral ceiling regions farther from the tower exhibited significantly lower doses, in between 5 and 25 mJ/cm^2^, indicating reduced UV‐C exposure in the most distant areas. However, no location recorded a dose of zero. These results align with findings from previous studies,^24^ which highlight that elevated surface, such as ceilings, often receive limited direct irradiation and are primarily exposed to reflected UV‐C light from surrounding surfaces. This indirect exposure results in lower radiation intensity and, consequently, diminished disinfection efficacy in such regions. The efficacy of UV‐C radiation on the ceiling observed in this study follows the trend where ceilings and elevated surfaces tend to be disinfected less efficiently due to the distance from the radiation source.^25^

Analysis of the dosimeters installed on the walls also revealed variations in UV‐C dose, following the same trend observed on the ceiling. Vertical surfaces closer to the lamp or positioned at a favorable angle for light reflection received adequate doses of germicidal radiation, reaching approximately 25 mJ/cm^2^. In contrast, areas less favored by direct radiation, such as shadow zones, presented lower doses, varying between 5 and 25 mJ/cm^2^. Studies indicate that vertical surfaces are frequently less exposed to direct UV‐C radiation, resulting in inconsistent doses.^6^

The results indicate that colorimetric films can serve as practical tools for real‐time dose monitoring, and that careful planning of exposure time and distance is essential to ensure the desired effectiveness of the equipment.

**FIGURE 7:**
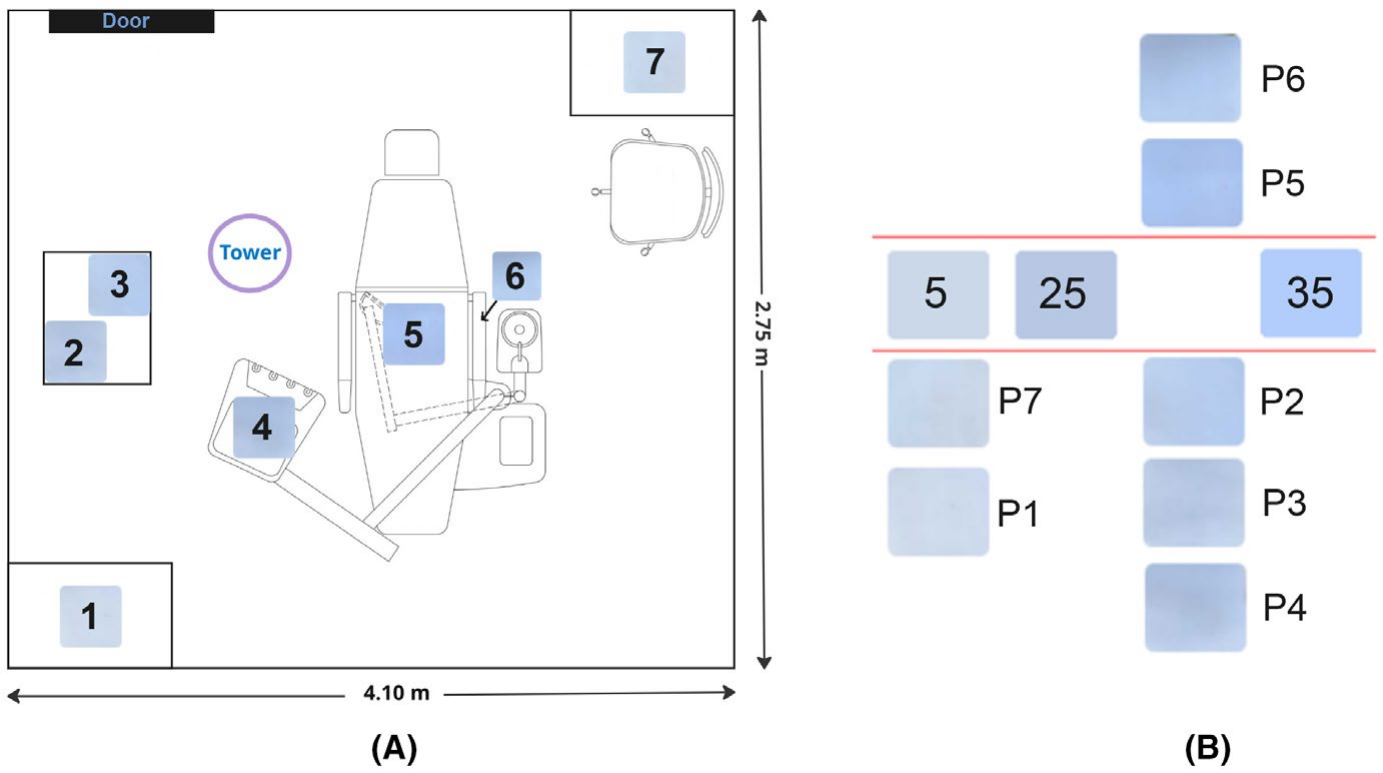
(A) Schematic layout of the dental operatory room (4.10 × 2.75 m) showing the positioning of the UV‐C disinfection tower (purple circle labeled “Tower”) and the seven microbiological sampling points (blue squares labeled 1 to 7). (B) Estimated UV‐C dose values (in mJ/cm^2^) derived from the colorimetric comparison with the reference template (center), providing quantitative assessment of the radiation absorbed at each sampled location.

Following the analyses of the ceiling and wall dosimeters, it was decided to position the tower near the dental chair (dental unit), since locations farther from the source exhibited low UV‐C exposure. For this test, the colorimetric films were placed horizontally at seven specific sites of clinical relevance, as shown in Figure 7A, the same locations where microbiological samples were collected before and after irradiation. Further details of the procedure were provided in Section “Microbiological sampling and UV‐C dose mapping with colorimetric films” of the Materials and Methods.

At Point 2 (located 0.60 m from the radiation source), Point 3 (0.70 m), Point 4 (0.70 m), Point 5 (0.80 m), and Point 6 (0.90 m), the measured doses ranged between 25 and 35 mJ/cm^2^, reflecting direct exposure to the UV‐C source (Figure 7B). Points 5 and 6 exhibited some inhomogeneity on the films, with visible bluish streaks, while Points 2, 3, and 4 demonstrated more uniform coloration, indicating a more homogeneous radiation distribution across the exposed area. The high incidence of radiation in these areas highlights the effectiveness of positioning the dosimeters on horizontal surfaces and their direct exposure to UV‐C light.

However, points such as Point 1 (1.90 m) and Point 7 (1.80 m) presented lower doses, between 0 and 5 mJ/cm^2^ (Figure 7B). These results highlight the importance of adjusting the arrangement of furniture in the office to allow UV‐C radiation to reach all surfaces effectively.

Despite the promising results, our study has some important limitations. All experiments were performed under controlled conditions in a single dental operatory, with a fixed tower position and a specific furniture layout, so the findings may not directly generalize to other rooms or workflows. In routine practice, an effective protocol would likely require combining appropriate furniture arrangement with multiple tower positions and/or longer exposure times to minimize shadowed regions. Our dose–response and dosimetric data also suggest that local colorimetric dosimetry can be used as a practical tool to identify underexposed areas where shading is most critical and where repositioning of the UV‐C source is required.

### In situ UV‐C evaluation at strategic points with microbial sampling

To assess the possible influence of swab collection on the effectiveness of UV‐C disinfection, the predetermined points were divided into two symmetrical halves, assuming a homogeneous distribution of microorganisms on the surfaces. At each sampling point, only one‐half of the surface was collected before UV‐C exposure, while both sides were collected after irradiation, allowing a paired comparison between previously collected and noncollected areas. It is important to highlight that all collections were performed after routine cleaning procedures, ensuring that the residual microbial load represented realistic postcleaning contamination conditions, as generally found in clinical practice. Further details of the procedure were provided in “Microbiological sampling and UV‐C dose mapping with colorimetric films” of the Materials and Methods.

The seven sampling points selected for this study were strategically determined based on two primary criteria:

(i) The diversity of surface materials commonly found in dental clinics and (ii) the frequency of contact with professionals, patients, or aerosol‐generating procedures during routine care. This careful selection aimed to reflect realistic contamination scenarios and allowed comparisons between high‐risk and low‐risk areas regarding microbial load and UV‐C exposure.

**FIGURE 8:**
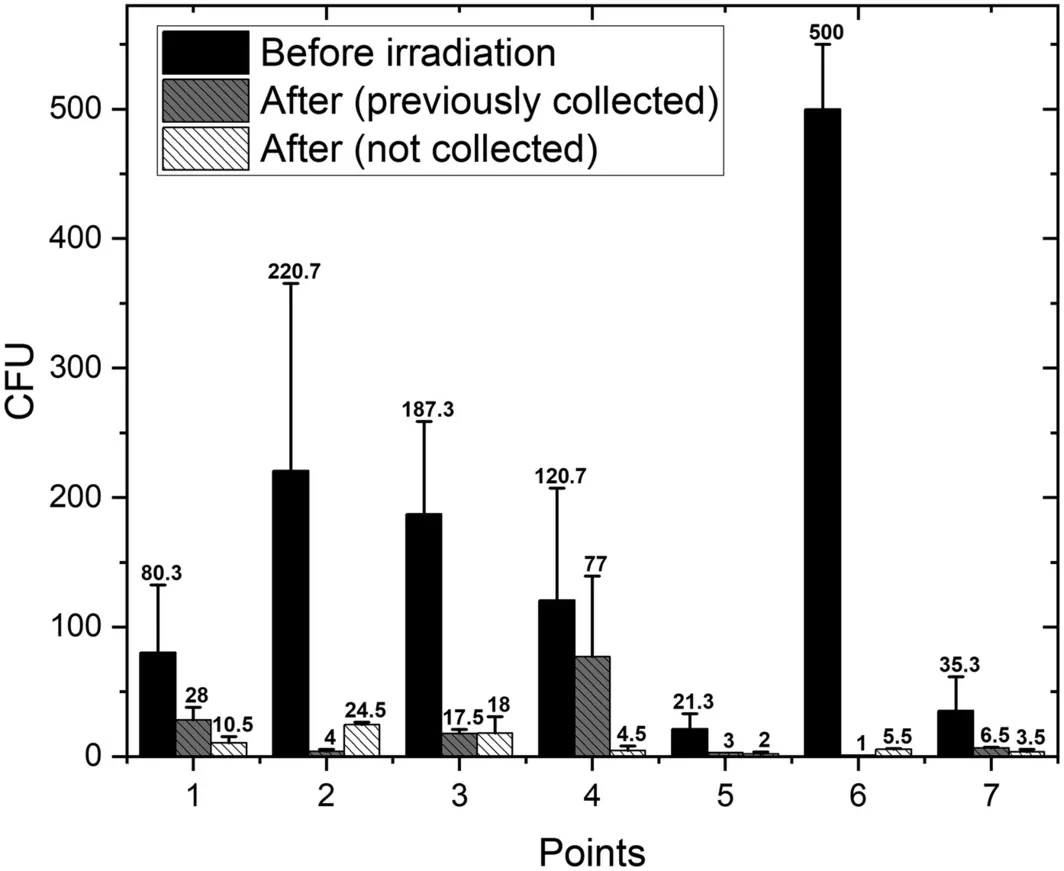
Mean colony‐forming units (CFU) at the seven sampling points before UV‐C irradiation (black bars) and after irradiation on the previously swabbed halves (dark hatched bars) and nonswabbed halves (light hatched bars). Numbers above the error bars indicate the mean CFU for each condition; error bars represent the standard deviation of the measurements.

Among these surfaces, the armrest of the dental chair stood out due to its high initial microbial load, averaging over 500 CFUs in triplicate before UV‐C application (as can be seen in Point 6 of Figure 8). Thus, the dental chair armrest (Point 6) exhibited an average pre‐UV‐C count of >500 CFU (measured after routine cleaning) and showed a reduction greater than 90% after UV‐C exposure (≈1‐log reduction).

Despite routine cleaning practices performed prior to collection, this site remains highly susceptible to recontamination due to direct handling and proximity to aerosol dispersion. Notably, even with such elevated baseline contamination, UV‐C exposure promoted a sharp microbial reduction, indicating that the system was effective even on critical surfaces prone to high bioaerosol deposition.

To formally assess whether these reductions were statistically significant across all locations, we compared the point‐wise mean CFU counts before and after UV‐C exposure using the Wilcoxon signed‐rank test. Across the seven sampling points, UV‐C irradiation produced significant reductions on both halves of the surfaces: “Before irradiation” versus “After irradiation (previously collected)” yielded *W* = 0, *p* = 0.0156, and “Before irradiation” versus “After irradiation (not collected)” likewise yielded *W* = 0, *p* = 0.0156.

**FIGURE 9:**
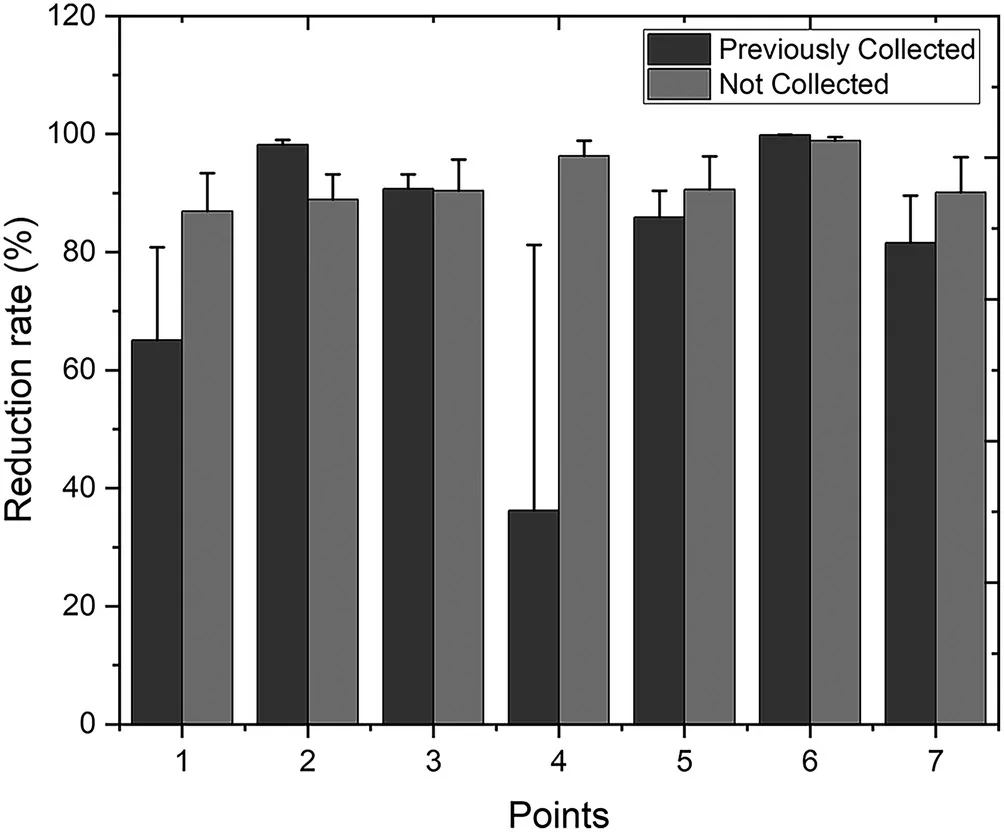
Mean reduction rate (%) at the seven measurement points (1–7) comparing previously collected samples (dark bars) and not collected samples (light bars). Error bars indicate the standard deviation.

Figure 9 summarizes these data as percentage reductions for the previously swabbed and nonswabbed halves of each sampling point. Importantly, preirradiation swabbing did not prevent effective UV‐C disinfection: both sides showed high reduction rates at all seven locations, with no sampling point dropping below ~70% reduction. Considering all positions, the mean CFU decrease was 79.6% ± 8.5% on the previously collected side and 91.7% ± 1.6% on the noncollected side, indicating only a modest reduction in performance associated with prior swabbing.

As these results are derived from the CFU counts in Figure 8, the Wilcoxon test confirmed the same statistically significant reduction in microbial load after UV‐C exposure (*W* = 0.0, *p* = 0.0156) for both conditions.

These findings confirm that the UV‐C protocol achieves a consistent and statistically significant reduction in microbial load at all evaluated locations, reinforcing the reliability of the disinfection device when appropriately positioned and operated under controlled exposure conditions. From a practical standpoint, they also indicate that routine swabbing before irradiation does not compromise the disinfection process.

Building on these microbiological results, subsequent analysis focused on correlating each point's UV‐C dose (as measured by the colorimetric dosimeters) with the distance to the source and the resulting microbial reduction (Table 1).

**TABLE 1:** Microbiological sampling results before and after UV‐C irradiation, with corresponding radiation dose (mJ/cm^2^) and distance from the light source (m).

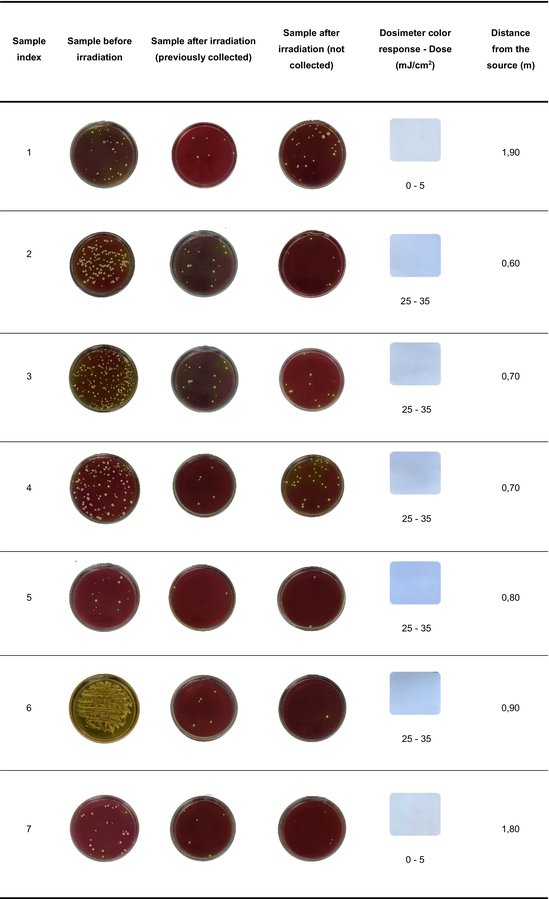

Points 2–6, located between 0.60 and 0.90 m from the disinfection tower, received doses between 25 and 35 mJ/cm^2^, sufficient to inactivate both *S. epidermidis* (10–20 mJ/cm^2^) and *S. aureus* (20–40 mJ/cm^2^), as reported in the literature.^23^, ^26^

Point 6, in particular, deserves attention. Although it was located on a textured or more geometrically complex surface, and initially presented a high microbial load, UV‐C exposure resulted in a marked decrease in CFU count, proving the system's effectiveness even in areas with structural characteristics less favorable to uniform irradiation.

By contrast, Points 1 and 7, positioned approximately 1.80 and 1.90 m from the source and receiving doses below 5 mJ/cm^2^, exhibited lower microbial reductions. These doses fall well below the thresholds required for effective staphylococcal inactivation,^26^ highlighting the need for proper equipment positioning and the importance of avoiding shaded or distant zones that limit UV‐C propagation.

The surface material also influenced the results. Smooth, nonporous, and more reflective materials such as stainless steel (Points 3 and 4) facilitated a more uniform superficial exposure to UV‐C, which is consistent with the homogeneous dosimeter coloration and higher microbial reduction observed at these points. This effect is attributed to reflectivity and surface regularity rather than any penetration of UV‐C into the material. In contrast, Points 5 and 6, though receiving similar doses, showed some uneven dosimetric patterns, likely due to surface irregularities. Nevertheless, Point 6 still achieved excellent microbial reduction, emphasizing that UV‐C is effective even on complex surfaces when the dose is sufficient. Microbiological analysis identified both *S. aureus* (mannitol‐fermenting) and *S. epidermidis* (nonfermenting), organisms commonly found in healthcare environments. After UV‐C application, both species showed notable reductions at sites with adequate radiation dose, confirming the protocol's efficacy against multiple types of staphylococcal strains. Since parameters tested impacted irradiation and antibacterial efficiency of UV‐C radiation, the null hypothesis was rejected.

Thus, the integration of dosimetric and microbiological data validated the germicidal effectiveness of the UV‐C tower. The correlation between distance, dose, and microbial reduction strengthens the potential of this system as a complementary tool to routine chemical disinfection in dental settings, especially when tailored to clinical spatial layouts.

Importantly, UV‐C was able to inactivate bacteria that remained viable even after conventional chemical cleaning, particularly on high‐contact surfaces such as dental chair armrests and adjacent furniture, which initially exhibited high microbial loads. The successful inactivation of *S. aureus* and *S. epidermidis* on different materials highlights its potential for practical application.

This study stands out for the development and evaluation of a UV‐C disinfection device, specifically designed for dental and hospital environments. While most of the literature emphasizes high‐power UV‐C systems,^24^ this work explores the feasibility of moderate‐power devices, which not only reduce energy consumption but also ensure safer operation in environments with complex geometries, such as dental offices. The device features optimized to ensure regarding UV‐C application in such spaces. This approach aligns with the growing demand for sustainable and safe disinfection solutions, particularly in dental contexts where UV‐C technology remains underutilized.

Finally, it is important to emphasize that UV‐C irradiation does not replace conventional chemical disinfection methods but serves as a complementary approach. The adoption of a moderate‐power system, with reduced energy consumption and enhanced safety, strengthens existing protocols, particularly in dental settings. Therefore, its integration into routine practice represents a viable and sustainable strategy to enhance biosafety without compromising established procedures, thereby promoting greater protection for both professionals and patients.

## CONCLUSIONS

This study demonstrated that the moderate‐power UV‐C disinfection tower is effective in reducing the microbial load on surfaces in dental environments, achieving reduction rates exceeding 90% at points receiving adequate doses. The correlation between dose, distance, and efficacy, established through colorimetric dosimeters and microbiological analysis, highlights the importance of designing and dimensioning UV‐C systems tailored specifically for dental offices projects.

## FUNDING INFORMATION

This work was supported by the PAPIN Program at the Federal University of Pelotas (UFPel); the Research Support Foundation of the State of Rio Grande do Sul (FAPERGS); the Oswaldo Cruz Foundation (Fiocruz) through the FAPERGS‐FIOCRUZ Call 12‐2022, Rede Saúde RS [Grant # 23/2551‐0000511‐5]; and the Secretariat of Science, Innovation and Technology of the State of Rio Grande do Sul (SICT‐RS), Grant No. 34/2023/FPE No. 3704/2023.

## CONFLICT OF INTEREST STATEMENT

The authors declare no conflicts of interest.

## Data Availability

The data that support the findings of this study are available from the corresponding author upon reasonable request.
